# Sea nettle jellyfish venom targets proteoglycans to cause cell death and pain

**DOI:** 10.1126/sciadv.aec7585

**Published:** 2026-09-02

**Authors:** Christopher E. Denes, Tian Y. D'Araujo, Felicity Chung, Man-Tat Lau, Adam Cole, Nicholas R. Casewell, Eivind A. B. Undheim, G. Gregory Neely

**Affiliations:** ^1^ The Dr. John and Anne Chong Lab for Functional Genomics, Charles Perkins Centre and School of Life and Environmental Sciences, The University of Sydney, Sydney, NSW 2006, Australia.; ^2^ Centre for Snakebite Research and Interventions, Liverpool School of Tropical Medicine, Pembroke Place, Liverpool L3 5QA, UK.; ^3^ Centre for Ecological and Evolutionary Synthesis, Department of Biosciences, University of Oslo, N-0316 Oslo, Norway.

## Abstract

Sea nettle jellyfish cause millions of painful stings annually with little known about how their venom works and no rational treatments available. Here, we perform a systematic analysis of sea nettle venom/host interactions. The venom shows dose-dependent cytotoxic activity in human cells, and this can be blocked by dual inhibition of apoptosis and necroptosis. Using whole-genome CRISPR screening, we identified human genes and pathways that modify venom action. The top gene cluster identified regulates proteoglycan biosynthesis. We show that exogenous heparin, a drug used clinically as an anticoagulant, blocks venom cytotoxicity at a physiologically relevant dose. This effect was therapeutic, inhibiting venom even 1 hour after exposure. In vivo, heparin protected against acute spontaneous pain, thermal hyperalgesia, and mechanical allodynia induced by venom. This provides the exciting possibility of repurposing heparin, a safe, commercially available drug, as a prophylactic or therapeutic to reduce the impact of sea nettle stings.

## INTRODUCTION

The Atlantic sea nettle (*Chrysaora quinquecirrha*) is a jellyfish native to the Atlantic coast of the United States of America that is responsible for millions of human stings every year (*1*, *2*). Though nonlethal, envenoming events cause instant cutaneous pain that peaks in intensity within a few minutes and, in more severe cases, produce a raised red rash (*3*). These symptoms follow the explosive release of venom from the nematocyst organelles situated within the nematocytes that line jellyfish tentacles (*4*). Comprising a complex mixture of molecules, sea nettle venom (SNV) is toxic to skin, heart, nervous, kidney, liver and blood tissue (*5*–*10*), and has lethal and dermonecrotic properties in animals (*5*, *11*, *12*). The current dominant treatment for stings is the application of a 50:50 slurry of baking soda and ambient water to inhibit any further nematocyst discharge (*13*), followed by gentle removal of any remaining tentacle to prevent secondary stinging events. There are no molecular-based rational treatments for Atlantic sea nettle envenoming, and this is in part because we do not fully understand the molecular interactions between jellyfish venom and target physiology.

Functional genomics is a powerful approach for revealing the molecular mechanisms underlying health conditions and for discovering new drug treatments (*14*–*17*). One such approach involves the use of CRISPR technology to induce gene knockouts on a genome-wide scale, allowing for pooled functional screening to identify the underlying mechanisms and potential therapeutic targets of a given process (*17*, *18*). Pools of knockout cells are then challenged with a selective pressure, such as a cytotoxic venom (*14*, *15*), to rapidly identify genes that, when disrupted, can promote sensitization or resistance to that pressure. As venoms target and leverage host pathways to achieve their physiologic effects, the identification of host proteins that either directly or indirectly influence venom activity can help us better understand how the venom works, or how to block it (*14*, *15*, *19*, *20*). Here, we describe the application of functional genomics to elucidate target machinery required for *C. quinquecirrha* jellyfish venom to kill cells and cause pain.

## RESULTS

### *C. quinquecirrha* venom is cytotoxic

**Fig. 1.**
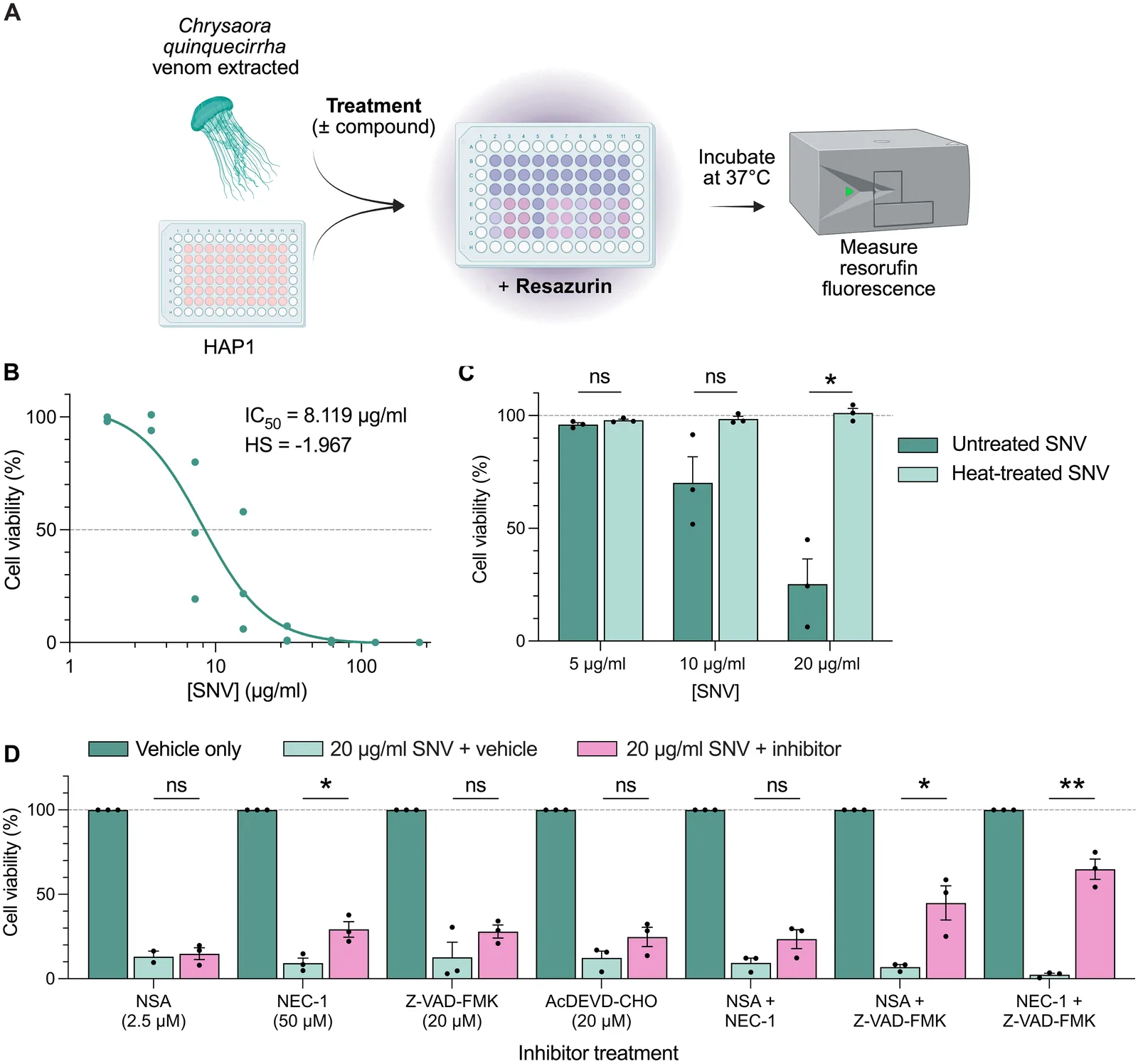
Programmed cell death pathways are necessary for SNV cytotoxicity. (**A**) Schematic of the resazurin reduction assay to test venom cytotoxicity. Created in BioRender. Neely, G. (2026) https://BioRender.com/kqds4vb. (**B**) HAP1 cell viability was measured by resazurin reduction assay 72 hours after treatment with SNV (*N* = 3). (**C**) HAP1 cell viability after exposure to heat-treated SNV. Data plotted are mean ± SEM (*N* = 3), with significance determined using an unpaired one-tailed parametric *t* test with Welch’s correction. (**D**) HAP1 cells were treated with inhibitors (NSA, NEC-1, Z-VAD-FMK, and Ac-DEVD-CHO) alone or in combination ± SNV (20 μg/ml). Cells were pretreated with inhibitors for 5 hours before SNV addition. Data plotted are mean ± SEM (*N* = 3), with significance determined using unpaired one-tailed parametric *t* tests with Welch’s correction.

To test whether SNV would be suitable for use with CRISPR screening, we first performed a dose-response evaluating cytotoxicity in human cells. HAP1 cells were treated with SNV for 72 hours and a resazurin reduction assay was used to determine viability (Fig. 1A). SNV was cytotoxic, with an IC_50_ of 8.119 μg/ml (Fig. 1B). Heat treatment destroyed SNV cytotoxic activity, even at a dose high enough to induce ∼80% lethality (20 μg/ml; Fig. 1C), indicating that the cytotoxic component is heat labile and thus likely protein in nature. To investigate the nature of SNV cytotoxicity, we treated cells with venom in the presence of inhibitors of apoptosis (AcDEVD-CHO and Z-VAD-FMK) or necroptosis [necrosulfonamide (NSA); necrostatin-1 (NEC-1)] (fig. S1). Alone, NEC-1 provided mild but significant protection from SNV (*P* = 0.0141, ∼3-fold increased cell viability), while combined inhibition of necroptosis and apoptosis provided strong protection (*P* = 0.0311, ∼6-fold higher viability for NSA + Z-VAD-FMK; *P* = 0.004, ∼27-fold higher viability for NEC-1 + Z-VAD-FMK; Fig. 1D).

### Whole-genome CRISPR knockout screen

**Fig. 2.**
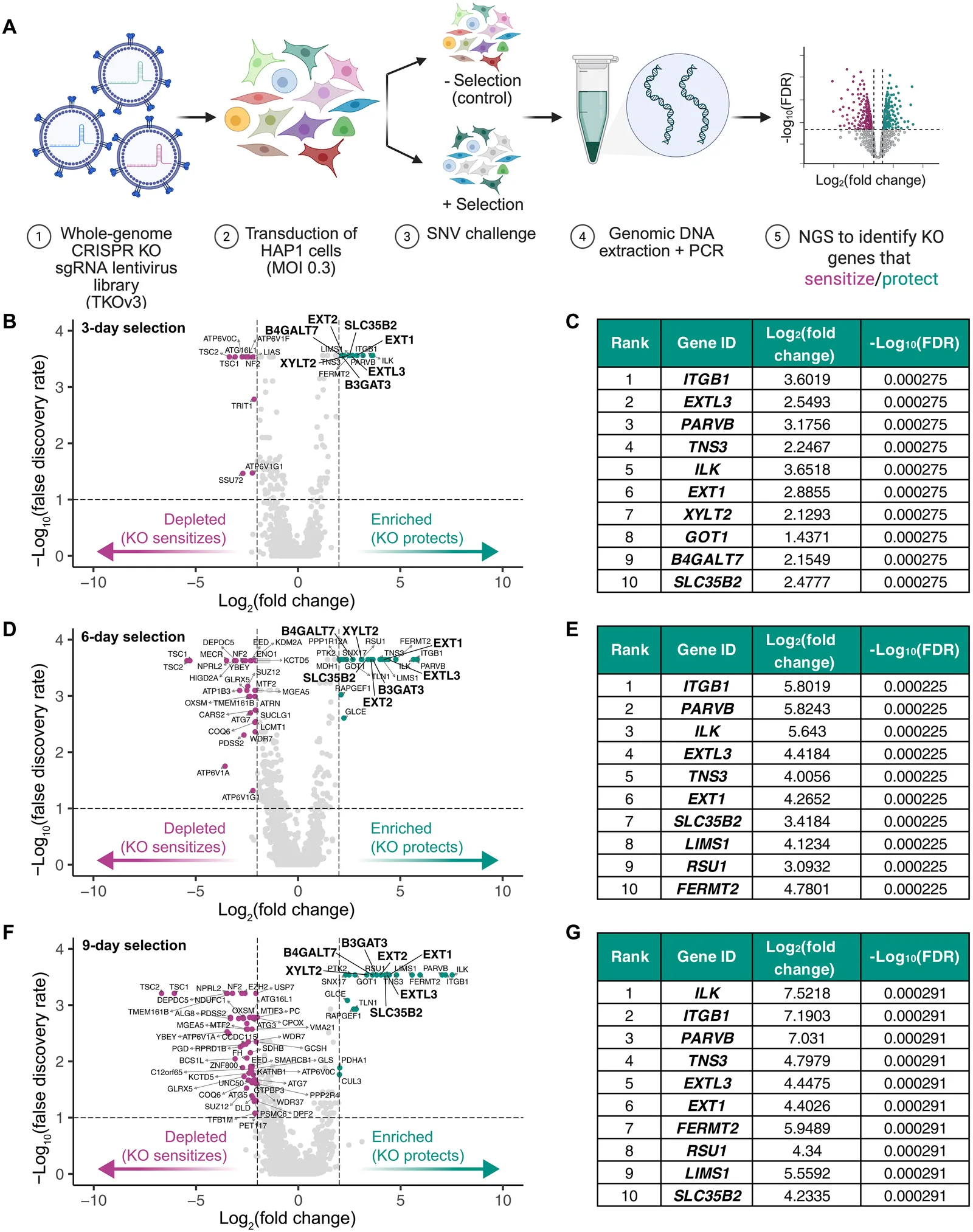
A whole-genome CRISPR knockout screen identifies enriched gene hits following venom selection. (**A**) Schematic of the workflow used for pooled whole-genome CRISPR knockout screening of SNV in HAP1 cells. Created in BioRender. Neely, G. (2026) https://BioRender.com/0x4qjal. Volcano plots portraying protective and sensitizing hits following 3 days (**B**), 6 days (**D**), and 9 days (**F**) of SNV selection (*N* = 2). The top 10 MAGeCK-ranked enriched gene hits following 3 days (**C**), 6 days (**E**), and 9 days (**G**) of SNV selection.

To gain insight into SNV cytotoxic mechanisms, we performed a whole-genome CRISPR knockout screen (Fig. 2A). Cells were transduced with a replication-deficient lentivirus library carrying the Toronto KnockOut CRISPR Library - Version 3 [TKOv3 (*21*)] at a multiplicity of infection (MOI) of 0.3, producing a pool of cells in which each cell harbors a single gene knockout. This pool was challenged with SNV (50 μg/ml; LD80) for up to 9 days against an unselected control, with surviving cell populations sampled every 3 days. The integrated sgRNA-containing sequence was amplified from isolated genomic DNA (gDNA) and sgRNA read counts determined by short-read Illumina sequencing. sgRNA enrichment or depletion in the selected population compared to the control population was assessed using MAGeCKFlute (*22*) (3-day selection, Fig. 2, B and C, and table S1; 6-day selection, Fig. 2, D and E, and table S2; 9-day selection, Fig. 2, F and G, and table S3). sgRNA hits that dropped out after SNV selection sensitized cells to venom [log_2_fold change (FC) < −2; −log_10_false discovery rate (FDR) > 1], while hits that were enriched postselection promoted venom resistance (log_2_FC > 2; −log_10_FDR > 1) (*14*). A total of 67 depleted and 24 enriched FDR- and log_2_FC-significant unique hits were identified in the screen.

**Fig. 3.**
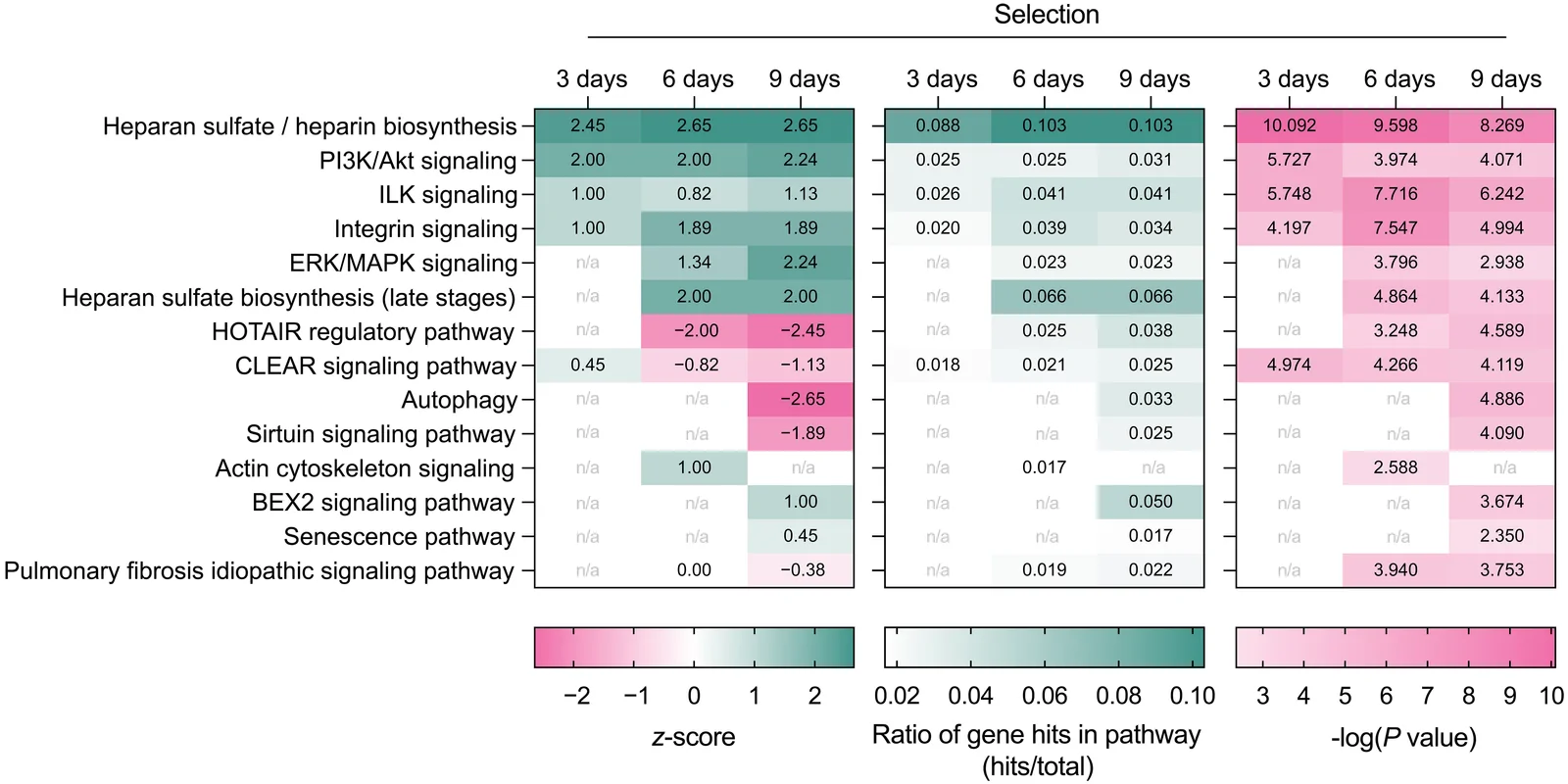
Pathway analysis identified heparan sulfate/heparin biosynthesis as the most enriched canonical pathway across all three screen time points. Significant gene hits (|log_2_FC| > 2 and −log_10_FDR > 1) from MAGeCKFlute were analyzed in Ingenuity Pathway Analysis. “n/a” indicates that the pathway was not identified.

Pathway analysis identified heparan sulfate/heparin biosynthesis, PI3K/AKT signaling, ILK signaling, and integrin signaling as enriched pathways at all three time points (Fig. 3). After 9 days of selection (Fig. 2G), 3 of the top 10 hits contributed to heparan sulfate/heparin biosynthesis: exostosin like glycosyltransferase 3 (EXTL3), an enzyme that catalyzes the addition of *N*-acetyl-d-glucosamine to glycosaminoglycan chains (*23*); exostosin glycosyltransferase 1 (EXT1), another glycosyltransferase critical to heparan sulfate/heparin chain elongation (*23*); and solute carrier family 35 member B2 (SLC35B2), a sulfotransferase that sulfates glycoproteins, glycolipids and proteoglycans (*23*). The other 7 hits in the top 10 contributed to integrin or integrin-linked kinase (ILK) signaling pathways or localization: ILK, a regulator of integrin-mediated signal transduction at the plasma membrane (*24*); the cell surface receptor integrin subunit beta 1 (ITGB1, also known as CD29) (*25*); the actin-binding protein beta parvin (PARVB) (*26*); fermitin family homolog 2 (FERMT2), a member of the extracellular matrix that regulates integrins (*27*); Ras suppressor protein 1 (RSU1), a protein that functions in Ras signal transduction but can also support integrin-mediated cell-cell adhesion (*28*); LIM zinc finger containing 1 (LIMS1), an adaptor protein within focal adhesions with functions in integrin signaling (*28*); and tensin 3 (TNS3), an anchor between integrins and the cytoskeleton regulator of focal adhesion (*29*). These top 10 genes were identified as significant hits after 3 and 6 days of selection as well, with only RSU1 below hit cutoffs (log_2_FC = 1.836) at the 3-day time point.

Because heparan sulfate/heparin biosynthesis was the most enriched canonical pathway at all three selection time points by *z*-score, ratio and −log(*P* value) metrics (Fig. 3), we prioritized this pathway for follow-up study.

### Heparan sulfate/heparin biosynthesis is essential for SNV cytotoxicity

**Fig. 4.**
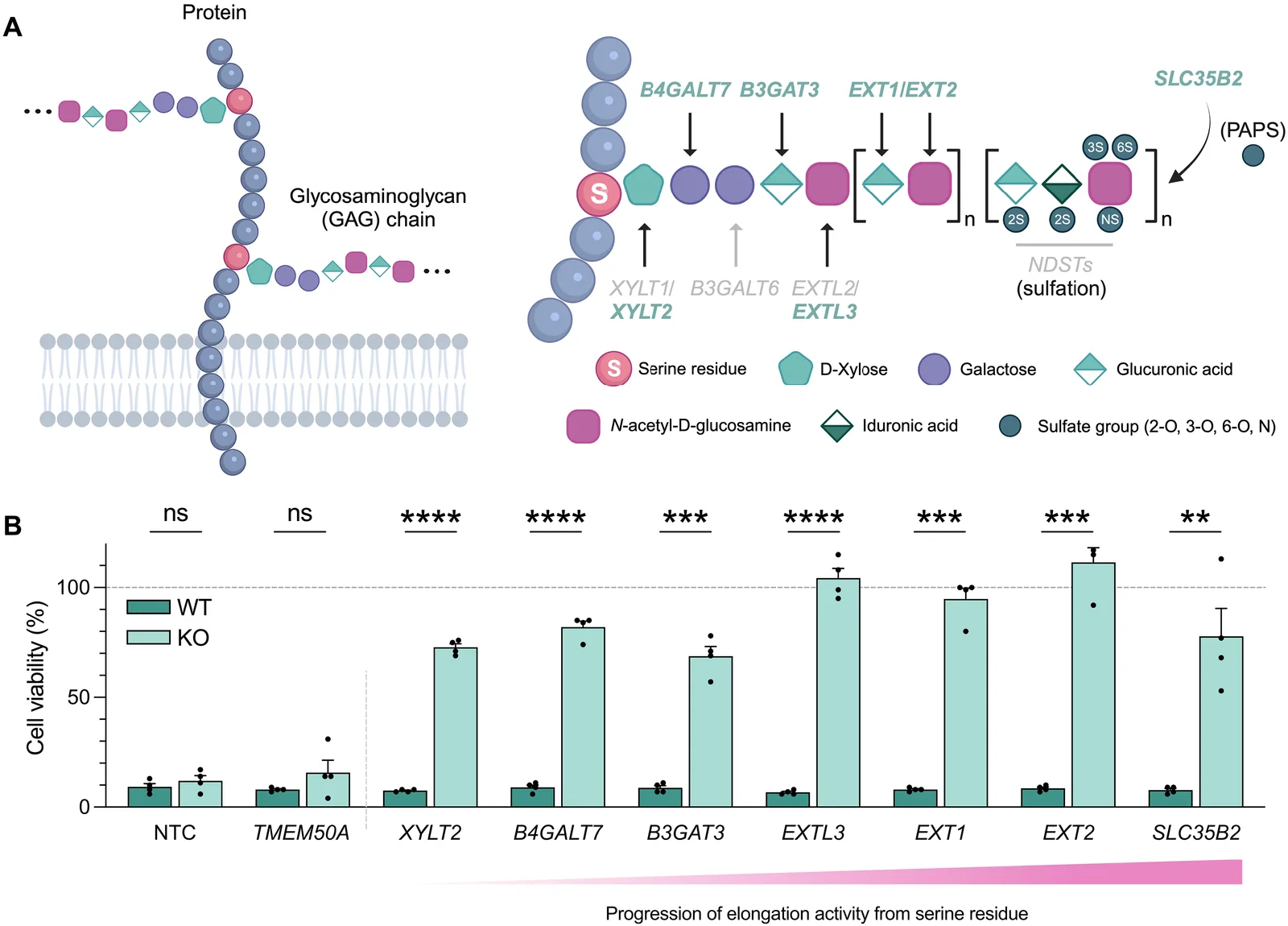
CRISPR knockout of heparan sulfate/heparin biosynthesis genes confers protection against SNV cytotoxicity. (**A**) Schematic representation of the heparan sulfate/heparin biosynthesis pathway. Pathway hits identified as significant in the screen are shown in bold green text; nonsignificant hits are depicted in gray. Panel modified from Du *et al.*, *Science Translational Medicine*, 10.1126/scitranslmed.adk4802 [2024], AAAS (*14*) (CCC RightsLink license number 6110640208289). Created in BioRender. Neely, G. (2026) https://BioRender.com/753h7m6. (**B**) Pools of single sgRNA knockout cells for nontargeting control (NTC), a negative on-target control (TMEM50A), and heparan sulfate/heparin biosynthesis gene hits were generated by lentiviral transduction. Wild-type (WT), NTC, and KO cells were treated with SNV (30 μg/ml) for 24 hours before viability was determined by resazurin reduction assay. Data plotted are mean ± SEM (*N* = 4), with significance determined using an unpaired one-tailed parametric *t* test with Welch’s correction.

Heparan sulfate proteoglycans are conserved across metazoans where they act as a scaffold for the extracellular matrix (*30*) and receptor ligand interactions across a myriad of cellular processes (*31*). Screen hits XYLT2, B4GALT7, B3GAT3, EXTL3, EXT1, EXT2, and SLC35B2 all contribute to heparan sulfate/heparin biosynthesis (Fig. 4A) and sit within the top 20 most significantly enriched protective knockouts at all time points (Fig. 2, B, D, and F, and tables S1 to S3). Heparan sulfate/heparin biosynthesis genes that did not reach statistical significance included the following: B3GALT6 (rank 142, FDR 0.326611), EXTL2 (rank 9105, FDR 1), XYLT1 (rank 10224, FDR 1), and the four NDSTs (NDST1 to NDST4) (all ranked >10,000, all FDRs 1) (9-day selection; table S3 and Fig. 4A).

To validate these hits, we generated knockout cell pools with single sgRNAs and evaluated whether disruption of each target gene rescued cell viability. Independent knockout of all seven pathway hits from the screen significantly improved cell viability post-SNV challenge (Fig. 4B). These data demonstrate that loss of the heparan sulfate/heparin biosynthetic pathway can result in significant inhibition of SNV cytotoxicity.

**Fig. 5.**
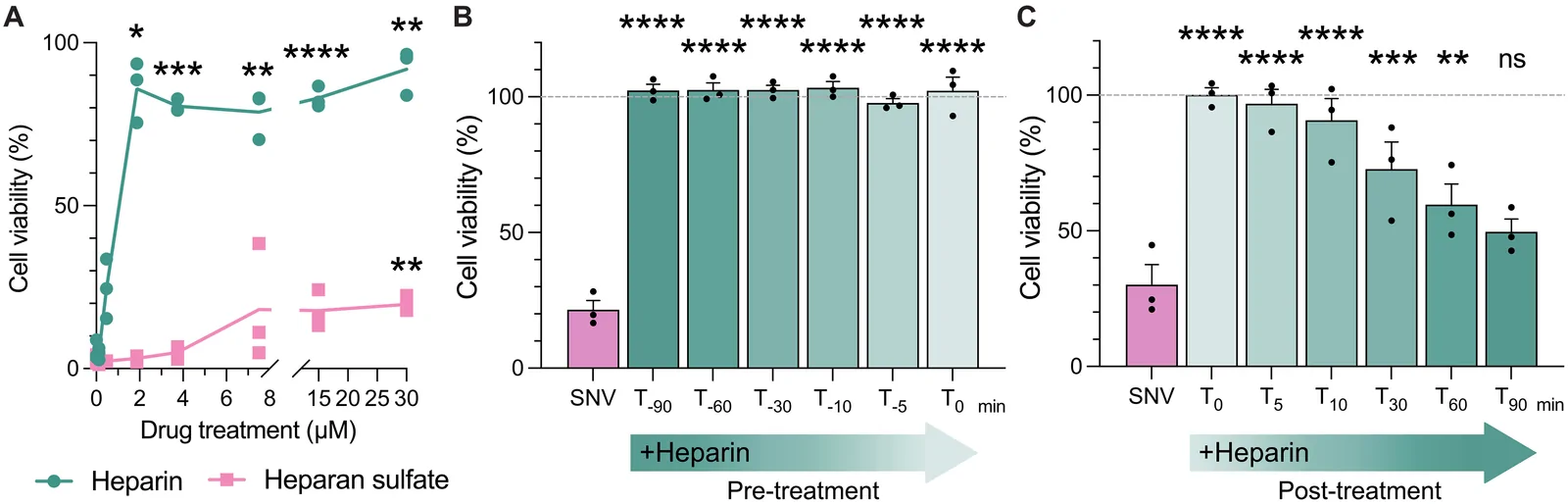
Supplementation of sulfated proteoglycans protects HAP1 cells from SNV cytotoxicity. (**A**) HAP1 cells were cotreated with sulfated proteoglycans and SNV (20 μg/ml) for 3 days and viability was assessed by resazurin reduction assay. Data plotted are mean ± SEM (*N* = 3) with significance determined using a one-way Welch’s ANOVA test with Dunnett’s T3 correction for multiple comparisons against vehicle conditions. Cell viability following pretreatment at 90, 60, 30, 10, and 5 min (**B**) or posttreatment at 5, 10, 30, 60, and 90 min (**C**) with 15 μM heparin before/after SNV (15 μg/ml) challenge. Data plotted are mean ± SEM (*N* = 3) with significance determined using a matched one-way ANOVA test with Dunnett’s correction for multiple comparisons with a single pooled variance against SNV-only conditions.

We next tested whether flooding the system with excess soluble heparan sulfate or heparin could act as a “decoy receptor” and suppress SNV cytotoxicity (*14*, *32*, *33*). We found that supplementation of culture medium with either polysaccharide could suppress SNV cytotoxicity, with heparin showing the most significant protection at a dose as low as 1.875 μM (*P* = 0.0159, Fig. 5A). This result raises the possibility of repurposing heparin, a Food and Drug Administration–approved WHO essential medicine (*34*), for the treatment or prevention of sea nettle envenoming. Pretreatment of cells with heparin for 0 to 90 min completely protects against SNV cytotoxicity (Fig. 5B), and a significant level of protection lasts even when heparin is added up to 60 min postenvenoming (*P* = 0.0039, Fig. 5C). Overall, these data suggest that heparin or related compounds may have utility as a prophylactic when at high risk of a sting, or therapeutically after envenoming.

### Heparin reduces pain from SNV in vivo

**Fig. 6.**
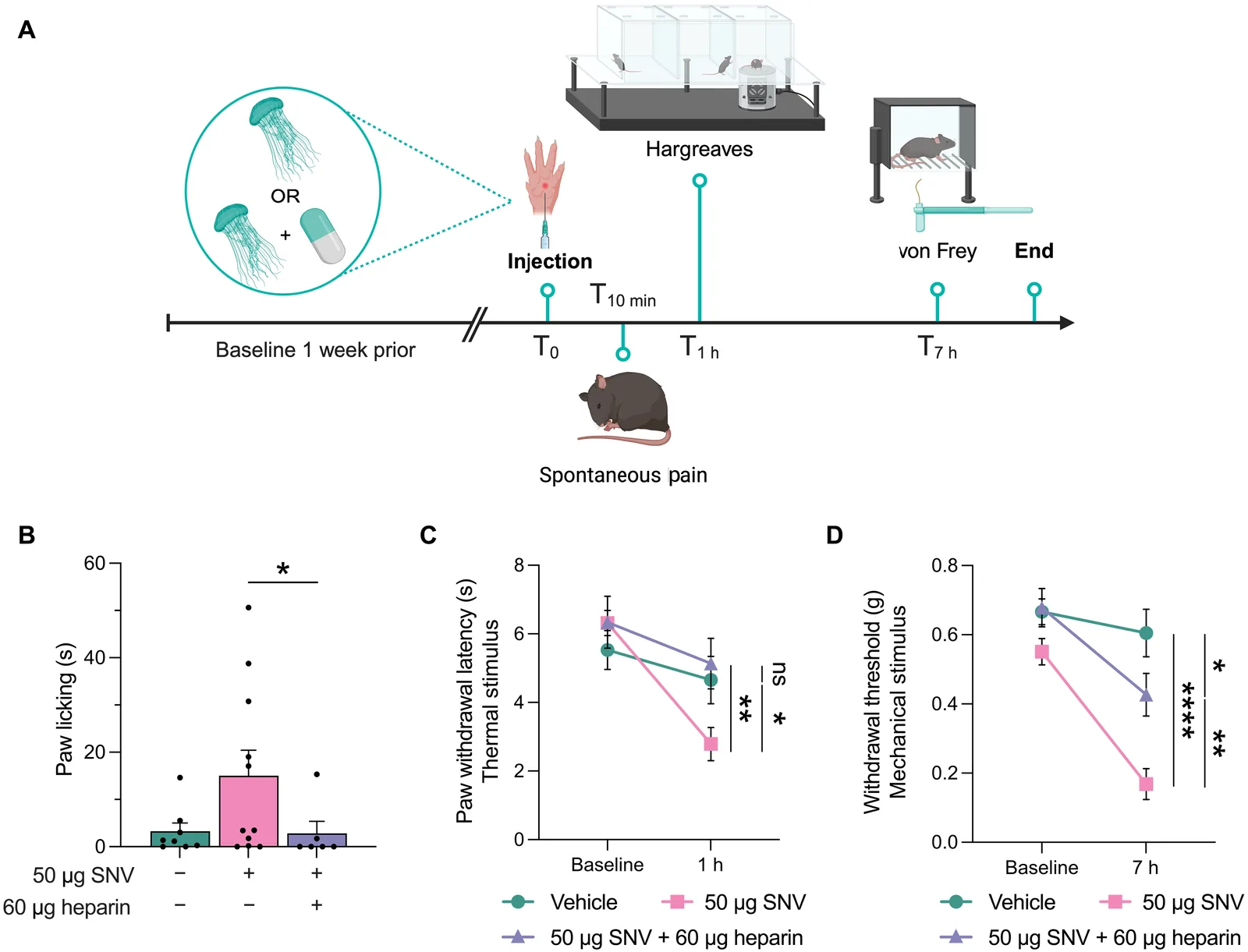
A footpad envenoming model highlights the protective effect of heparin in vivo. (**A**) Schematic representation of the footpad envenoming model and time course of nocifensive behavior evaluation. Young male C57BL/6 mice were injected in the rear left footpad with 25 μl of vehicle (20 mM Hepes buffer) or 25 μl carrying 50 μg of SNV or both 50 μg of SNV and 60 μg of heparin. Nocifensive behaviors were recorded up to 7 hours postinjection. Created in BioRender. Neely, G. (2026) https://BioRender.com/s6mhnfp. (**B**) Spontaneous paw licking responses were recorded for the first 10 min postinjection. Data plotted are mean ± SEM, with significance determined using a one-tailed Mann-Whitney test. (**C**) Withdrawal responses to a thermal stimulus were measured 1 hour postinjection using a Hargreaves assay. Data plotted are mean ± SEM with significance determined using a two-way ANOVA with Fisher’s LSD test. (**D**) Withdrawal responses to a mechanical stimulus were measured 7 hours postinjection using a von Frey test. Data plotted are mean ± SEM with significance determined using a two-way ANOVA with Fisher’s LSD test. For (B) to (D), *N* = 8 for vehicle, *N* = 11 for SNV alone, and *N* = 6 for SNV + heparin.

We next tested whether heparin could provide protection from sea nettle envenoming in vivo, adapting WHO-recommended 3R-refining protocols for finding the minimum required dose for evaluating envenoming treatments (*14*, *35*). We first developed behavioral assays to evaluate pain in response to sea nettle envenoming (Fig. 6A). After venom injection in the footpad, there were no obvious signs of inflammation (paw swelling or redness); however, mice exhibited an acute SNV-evoked spontaneous nocifensive response involving paw licking, and coadministration of heparin (pre-mixed with venom) reduced this immediate response, returning the response to vehicle-only levels (Fig. 6B). One-hour postinjection, mice treated with SNV exhibited thermal hyperalgesia (where noxious heat sensitivity is increased; assessed by Hargreaves test), and again heparin blocked this effect (Fig. 6C). This response was transient, with hypersensitivity normalizing by 6 hours. Last, SNV injection caused a late-stage mechanical allodynia (where an innocuous touch stimulus becomes painful; assessed by von Frey test), and heparin treatment reduced this pain sensitization (Fig. 6D). Together, these data show that heparin can reduce pain caused by sea nettle stings.

## DISCUSSION

Using an unbiased functional genomics approach, we found multiple venom-target genetic interactions required for SNV to harm human cells and characterized the interaction of one contributing pathway, heparan sulfate/heparin biosynthesis, in detail. Furthermore, we showed that heparin could be used to prevent venom cytotoxicity in vitro and the pain associated with envenoming in preclinical mouse models. Overall, this work has improved our basic molecular understanding of SNV/target interactions, and we used this knowledge to develop a therapeutic strategy to treat sea nettle stings.

Early molecular studies of *C. quinquecirrha* venom used isoelectric focusing to isolate distinct dermonecrotic, lethal, contractile, and fibrinolytic elements from SNV (*5*, *6*). Various studies have since determined the toxicity of SNV or fractions therein to skin, heart, nervous, kidney, liver, and blood cells or tissue (*7*–*10*). Purified venom can induce the formation of monovalent ion channels in both artificial lipid membranes and frog neuronal plasma membranes (*36*, *37*). In cardiac skeletal muscle, a component within the venom mixture triggers the formation of a monovalent sodium channel, affecting cardiac action potentials (*38*). The clinical symptoms of envenoming are shown to be in part induced by complement activation and subsequent cell death by lysis or apoptosis (*12*). In addition, in a report of an anaphylactic patient, purified venom was found to trigger strong histamine release from isolated basophils (*39*). Molecular work on the related *Chrysaora fuscescens* jellyfish has characterized the venom proteome (*40*) and reported the capacity for jellyfish venom to uncouple oxidative phosphorylation and induce an atypical apoptosis-like cell death (*41*). Despite these studies, therapeutic treatments for stings remain sparse, although topical lidocaine, an anesthetic that inhibits voltage-gated sodium channels, has been shown to minimize the number of discharging nematocysts for both *C. quinquecirrha* and *Chrysaora chinensis*, while only reducing pain for *C. quinquecirrha* (*42*, *43*).

Our whole-genome study reported here highlights the importance of heparan/heparin in SNV/human target interactions. Heparan sulfate biosynthesis is conserved across metazoans (*44*) and these glycosaminoglycans are essential for life (*45*–*47*). Various distinct structural motifs in the repetitive heparan sulfate polysaccharide chains act as binding sites for protein ligands, and these are additionally attractive for their heavily negative charge (*48*). Because of its broad expression and attractive binding characteristics, heparan sulfate has been adopted as a core cellular entry point for multiple viruses (*49*–*57*) and bacteria (*58*, *59*), also supporting the binding and internalization of various bacterial toxins (*60*–*62*). Proteinaceous venom toxins have also been found to interact with these proteoglycans; for example, the Chinese cobra (*Naja atra*) venom cardiotoxin uses heparan sulfate to increase its penetrance into membranes (*63*, *64*). Recently, we have independently identified heparan sulfate as a cellular target required for the cytotoxic effects of cobra venoms (*14*). Together, heparan sulfate may represent a core and convergent entry point for biologics arising from multiple kingdoms.

The synthesis pathways of heparan and heparin rely on the same core enzymes. Heparan sulfate is particularly important in the basement membrane between the epidermis and dermis, where it plays a role in the structural integrity and barrier function of our skin (*65*). In contrast, heparin is a core component of tissue-resident mast cell and basophil secretory granules (*66*) and is released from mast cells upon degranulation as part of a rapid innate immune response to biological threats (*67*). Heparin chains are more heavily sulfated than heparan sulfate, resulting in a highly negative charge that stabilizes mast cell granule proteases, and on release acts to modulate inflammation, block coagulation, and inhibit extracellular protease activities (*68*). This activity has been linked to enhancing resistance to venoms and other environmental threats such as the bee toxin melittin (*69*, *70*), some mammalian metalloproteases (*71*), and Gila monster and scorpion venoms (*72*). While the biological role for heparin is to stabilize secretory granule components and act as a direct anticoagulant (*73*, *74*), our work and others (*72*, *75*) suggest that it may also have primordial innate immune activity in its own right. Mechanistically, because heparin is more negatively charged than heparan, extracellular free heparin may directly bind to cationic toxins and block their ability to bind cell surface heparan sulfate, thereby blocking toxin action.

Beyond venom and toxins, other applications could use systemic delivery of heparin (or non-anticoagulating analogs) to inhibit the binding of viruses and bacteria in the body to heparan sulfate proteoglycans and their subsequent entry into cells. Already, affinity apheresis using Seraph 100 Microbind Affinity Blood Filters (a medical device containing beads covalently bonded to heparin) has been shown to deplete the levels of methicillin-resistant *Staphylococcus aureus* (*76*), herpes simplex virus type 2 (*77*), and SARS-CoV-2 (*78*) from patient blood, among other heparan sulfate–binding organisms (*79*). However, this could also affect the levels of cytokines and growth factors that drive or prevent inflammation or otherwise modulate disease (*80*). Future in vivo testing of heparin derivatives, like the low–molecular weight heparinoids tinzaparin and dalteparin used in our cobra study (*14*), or even heparan sulfate mimetic polymers (*81*), would be valuable in pushing forward the translation of heparinoids as broad-spectrum therapeutics.

Beyond heparin, our knockout screen also identified integrin- and integrin-linked signaling as top enriched pathways of interest. Integrin complexes form the transmembrane bridge between the extracellular matrix and the intracellular cytoskeleton. These complexes are critical in cell-cell adhesion and can transduce both biochemical and mechanical signals for the cell. The requirement for integrin signaling in SNV cytotoxicity opens new mechanistic questions. For example, does a component of the venom directly target and engage integrins to alter signaling and promote cell death? Or is this a more complex and indirect interaction? Many unrelated venom toxins are known to disrupt integrin dynamics. Venom disintegrins, derived by the proteolytic processing of P-II class snake venom metalloproteinases (SVMPs), can potently bind to targeted integrins through multiple recognition motifs (*82*), inhibiting cell adhesions and disrupting signaling. For example, the SVMP jararhagin from *Bothrops jararaca* disturbs integrin-regulated focal adhesion signaling, leading to reduced cell anchorage and apoptotic cell death (*83*). Such integrin recognition motifs have also evolved in viruses: an Arg-Gly-Asp motif in the ACE2 receptor-binding domain of the SARS-CoV-2 spike protein interacts with integrin αvβ1 for ACE2-mediated entry (*84*). Therapeutically, these toxin-integrin interactions are potentially druggable with competitive peptide mimetics or disintegrin-like molecules, akin to the development of the cyclic KGD peptide Integrilin (derived from the *Sistrurus miliarius barbouri* snake venom disintegrin barbourin) as an antithrombotic molecule.

Our screen also implicates an SNV-integrin interaction through the PI3K/Akt signaling pathway. ILK, ITGB1, LIMS1, and PTPA all sit upstream of Akt activation, a driver of cell proliferation/survival signals. Knockout of these genes conferred resistance to SNV cytotoxicity. This suggests that the venom exploits these proteins or their downstream signaling to induce cell death. In contrast, we found two depleted hits, TSC1 and TSC2, that also regulate this pathway. These proteins form a complex that can inhibit mTORC1 and, under stress conditions, signals through mTORC2 to activate Akt (*85*). The loss of either gene sensitized cells to venom, signifying that SNV induces cellular stress that is typically buffered by TSC-dependent signaling. Multiple other venoms have been shown to modulate the PI3K/Akt signaling pathway (*86*–*92*), frequently implicated in changes to cell motility, migration, and apoptosis. Though we prioritized validation of the heparan sulfate/heparin biosynthesis pathway in our study of SNV mechanism of action, the contribution of other genes and pathways identified here reveal additional vulnerabilities in our defense system that biologics have exploited.

Our study does have limitations. There are multiple strategies to isolate and store SNV that may affect the molecular pathways involved in SNV cytotoxicity (*93*). Here, we have used lyophilized venom (*11*, *94*, *95*), which in other studies has shown comparable activity to freshly isolated venom (*93*). However, a direct comparison between fresh and lyophilized venom would further strengthen the mechanisms described here. Though we investigated the venom of *C. quinquecirrha*, it would be interesting to see whether the related *C. chesapeakei* venom, only recently defined as an independent species of jellyfish (*96*), shows the same host requirements for its activity. Even within the same species, geographic distribution has been shown to contribute to differing venom characteristics (*97*). Last, in this study, we have validated 7 of the 91 significant screen hits (67 depleted and 24 enriched), and mechanistic validation of other hits within significant pathways will form the basis of future studies. Future in vivo studies should be performed to better understand the therapeutic window of new treatments postenvenoming. As SNV can induce ion channel formation that drives changes in action potential across membranes (*36*–*38*) and can stimulate mast cell histamine release that can drive itch and pain sensations (*39*), SNV-induced pain may rely on multiple independent mechanisms.

In summary, whole-genome CRISPR screening is a powerful functional genomics tool that enables the rapid identification of genes relevant to phenotypes of interest (*98*). Our study has identified the products of heparan sulfate/heparin biosynthesis as necessary for the cytotoxic activity of *C. quinquecirrha* venom. The WHO essential medicine heparin can block SNV activity and may have use in treating sea nettle stings.

## MATERIALS AND METHODS

### Cell culture

HAP1 and HEK293T cells were grown in Iscove’s modified Dulbecco’s medium (Sigma-Aldrich) and Dulbecco’s modified Eagle’s medium (Gibco), respectively, supplemented with 10% fetal bovine serum (Hyclone, no. SH30084.03), penicillin (100 U/ml), and streptomycin (100 μg/ml; Gibco, no. 15140122) and maintained in a humidified 37°C (5% CO_2_, atmospheric O_2_) environment. When passaging (before reaching ∼90% confluency), cells were rinsed with Dulbecco’s phosphate-buffered saline (Sigma-Aldrich, no. D8537) and trypsinized using TrypLE Express (Gibco, no. 12605-028).

### *C. quinquecirrha* venom

Lyophilized venom from *C. quinquecirrha* (referred to herein as SNV) was purchased from Sigma-Aldrich (no. V2502) and reconstituted in sterile, nuclease-free water and stored at −80°C in aliquots to minimize freeze-thaw. Venom was collected and extracted as previously described (*11*, *94*, *95*). Briefly, tentacles from adult jellyfish were homogenized to release nematocysts and tentacle debris removed by centrifugation. Nematocysts were then sonicated to release active venom. For thermolability testing, SNV was heat treated at 100°C for 5 min in a preheated heat block (*12*, *99*). *C. quinquecirrha* venom has proteolytic activity, which can rapidly degrade its cytotoxic potential (*94*), and so reconstituted SNV aliquots were only freeze-thawed once, with batch-specific dose responses determined.

### Chemicals

*N*-acetyl-Asp-Glu-Val-Asp-CHO (AcDEVD-CHO; no. S7901), NSA (no. S8251), and NEC-1 (no. S8037) were purchased from SelleckChem. *N*-benzyloxycarbonyl-Val-Ala-Asp(O-Me) fluoromethyl ketone (Z-VAD-FMK; no. V116), heparin sodium salt (no. H3393), and heparan sulfate (no. H7640) were purchased from Sigma-Aldrich. AcDEVD-CHO, heparin sodium salt, and heparan sulfate were reconstituted in sterile water. NSA, NEC-1, and Z-VAD-FMK were dissolved in sterile DMSO.

### Cell viability by resazurin reduction assay

Wild-type HAP1 cells were seeded into 96-well black plates with clear bottoms at a density of 3.5 × 10^4^ cells per well in 100 μl of growth medium. After 24 hours, growth medium was removed and cells were challenged with SNV at indicated doses. Cells were incubated with SNV for up to 72 hours before viability determined via a resazurin reduction assay. Medium was removed and resazurin (30 μg/ml; Sigma-Aldrich, no. R7017)–supplemented growth medium was added to wells and incubated at 37°C for up to 2 hours. Well fluorescence was measured using the 544/590 excitation/emission settings on a FLUOstar Omega plate reader (BMG Labtech). Viability was calculated from background-subtracted fluorescence values, comparing treatments to vehicle-treated control cells (representative of 100% viability).

For experiments with programmed cell death inhibitors, HAP1 cells were pretreated with indicated drugs for 5 hours before challenging with SNV. For rescue experiments with polysaccharide supplementation, cells were simultaneously treated with chemical and SNV, or pre-/posttreated with heparin up to 90 min before or after SNV challenge, respectively. For single gene knockout validation experiments, knockout cell pools were treated with SNV for 24 hours before transitioning to resazurin-supplemented medium for viability testing.

### CRISPR KO sgRNA library expansion

The Toronto KnockOut CRISPR Library—Version 3 (TKOv3) [Addgene, no. 90294 (*21*)] contains ∼71,000 sgRNAs cloned into a lentiCRISPRv2 vector, targeting each gene in the human genome with four distinct gRNAs (*21*). Pooled library DNA was amplified by expansion in *Escherichia coli* following electroporation. Briefly, 400 ng of plasmid DNA was electroporated into MegaX DH10B T1 R Electrocomp cells (Invitrogen, no. C640003) and grown at 30°C for 16 hours on Luria-Bertani agar (Invitrogen, no. 22700025) supplemented with carbenicillin (100 μg/ml; Thermo Fisher Scientific, no. J61949.14). Colonies were counted to ensure appropriate library coverage (minimum 500×) and plasmid DNA was isolated with the PureYield Plasmid Maxiprep System (Promega, no. A2393).

### TKOv3 library lentivirus production and titration

To produce lentivirus, HEK293T cells were seeded at 2 × 10^7^ cells per T175 flask and incubated overnight. Once at 70 to 90% confluency, cells were transfected using Lipofectamine 3000 (Invitrogen, no. L3000075) with a 1:3:3 ratio of pCAG-VSVG (Addgene, no. 35616):psPAX2 (Addgene, no. 12260):TKOv3 library. At 16 hours posttransfection, medium was refreshed. Lentivirus-containing supernatant was harvested 48 hours later, filtered through a 0.45-μm filter and concentrated 10× with a Pierce Protein Concentrator PES, 100K MWCO (Thermo Fisher Scientific, no. 88532). Lentivirus samples were stored at −80°C in single-use aliquots to minimize freeze-thaw.

The concentrated lentivirus library was titrated by transduction of HAP1 and subsequent viability testing after selecting for integration events. A total of 3 × 10^4^ HAP1 cells were seeded in 24-well plates overnight. A serial dilution of lentivirus, alongside untreated controls, was prepared in medium supplemented with polybrene (8 μg/ml; Sigma-Aldrich, no. H9268). Growth medium was removed and the lentiviral inoculum was gently applied to cells and incubated for 24 hours. Viral medium was then replaced with normal growth medium to let cells recover for 24 hours. Puromycin (Gibco, no. A1113803)–supplemented medium (1 μg/ml) was added to transduced cells to select for 72 hours. A resazurin reduction viability assay was performed to determine cell viability. Growth medium supplemented with resazurin (30 μg/ml; Sigma-Aldrich, no. R7017) was added to wells and incubated at 37°C for up to 4 hours. Supernatants were transferred to black 96-well plates with clear bottoms in triplicate, where well fluorescence was measured using the 544/590 excitation/emission settings on a FLUOstar Omega plate reader (BMG Labtech). Batch-specific viral volumes necessary for an MOI of 0.3 (corresponding to 30% cell survival) were calculated from background-subtracted fluorescence values, comparing surviving transduced cells to nontransduced control cells (representative of 100% viability).

### Library transduction and screening

Two independent replicates of 7 × 10^7^ HAP1 cells were transduced with lentivirus in medium supplemented with polybrene (8 μg/ml) at an MOI of 0.3 to achieve ∼300-fold coverage of the library after selection. After 24 hours, lentiviral media was removed and replaced with normal media to recover for another 24 hours. Cells were then selected with puromycin-supplemented medium (2 μg/ml) for 7 days.

Per replicate, pooled transduced HAP1 cells were then split into five T175 flasks (1.9 × 10^7^ cells per flask) and incubated overnight. Medium was replaced the next day with SNV (50 μg/ml)–supplemented medium for four flasks or normal medium for the fifth flask as a control. Cells were incubated with venom for 72 hours (primary selection) and then permitted to recover in normal growth medium for 72 hours. Cells were replated to correct for density and treated again with venom-containing media (second selection). This process was repeated for three total rounds of selection, with cells collected at each passage for gDNA analysis (8 × 10^7^ cells for controls and 4 × 10^7^ cells for selected treatments). Cell pellets were stored at −80°C until processing for all time points could be performed in parallel.

### gDNA sequencing

Frozen cell pellets were thawed, and gDNA was extracted with the ISOLATE II Genomic DNA Kit (Bioline). sgRNA-containing sequences were amplified from gDNA with nested PCR. For the first PCR reaction, gDNA for each sample (25 μg for controls and 5 μg for selected samples) was used as template. In 100-μl reaction volumes, 0.4 μM of each primer (NGSCRISPRv2F1 5′-GGACAGCAGAGATCCAGTTTGGT-3′; NGSCRISPRv2R1 5′-GAGCCAATTCCCACTCCTTTCAA-3′) was combined with 5 μg of gDNA and NEBNext High-Fidelity 2× PCR Master Mix (NEB, no. M0541) at a final 1× concentration, necessitating multiple 5-μg reactions to cover diversity controls. Thermocycling was performed in a Mastercycler nexus (Eppendorf): 98°C for 90 s; 20 cycles of 98°C for 10 s, 66°C for 30 s, and 72°C for 30 s; and 72°C for 120 s. After thermocycling, samples were pooled as relevant before a second PCR was performed to barcode samples and attach Illumina adapters. In 50-μl reactions, this PCR was performed with 10 ng of amplicon product from the first PCR, 0.4 μM of each primer, and NEBNext High-Fidelity 2× PCR Master Mix (NEB) at a final 1× concentration. Forward P5 staggered primer pool: 5′-AATGATACGGCGACCACCGAGATCTACACTCTTTCCCTACACGACGCTCTTCCGATCT[n]TCTTGTGGAAAGGACGAAACACC-3′ where [n] is a 1- to 7-nucleotide sequence, combined equimolarly; reverse P7 index-barcoded primer: 5′-CAAGCAGAAGACGGCATACGAGAT[index]GTGACTGGAGTTCAGACGTGTGCTCTTCCGATCTACCGACTCGGTGCCACTTTTTCAAG-3′ where the [index] is a 6-nucleotide barcode uniquely attributed to each sample. Thermocycling for this second PCR was performed in a Mastercycler nexus (Eppendorf): 98°C for 90 s; 3 cycles of 98°C for 20 s, 66°C for 30 s, and 72°C for 30 s; 15 cycles of 98°C for 20 s and 72°C for 30 s; and 72°C for 120 s. Amplicons were resolved by gel electrophoresis, isolated using the ISOLATE II PCR and Gel Kit (Bioline, no. BIO-52060) and quantified on a Qubit 3.0 fluorometer with the Qubit dsDNA HS Assay Kit (Thermo Fisher Scientific, no. Q32854). Barcoded amplicons were pooled and sequenced by NovogeneAIT Genomics Singapore using Illumina short-read sequencing. Short read whole-genome CRISPR KO sequencing datasets (FASTQ files) and related analysis files have been deposited at the Gene Expression Omnibus (GSE305962).

### CRISPR screen analysis

sgRNA enrichment and depletion in SNV-selected samples was evaluated and visualized using MAGeCKFlute (*22*). Read counts were compared pre- and post-SNV selection, using −log_10_FDR > 1 (i.e., FDR < 0.1) and |log_2_FC| > 2 cutoffs (*14*). Enriched/depleted genes were plotted against these thresholds using EnhancedVolcano (Version 1.10.0, R package; https://github.com/kevinblighe/EnhancedVolcano). Ingenuity Pathway Analysis [version 01-23-01 (*100*)] was implemented for identification of significantly enriched or depleted canonical pathways using gene inputs restricted to |log_2_FC| > 2 and FDR < 0.1. For comparison analyses, a *P* value cutoff of 0.05 was applied.

### Single gene KO validation

Top-performing sgRNAs from the TKOv3 library were introduced into the CRISPR knockout vector lentiCRISPRv2 (Addgene, no. 52961) (*101*) by restriction enzyme cloning to validate screen hits, alongside a nontargeting control sgRNA and a targeting negative control sgRNA for TMEM50A (for which no observed sensitization or protection was observed in the screen), referred to as NEG. Genes validated in this study overlapped with those identified in (*14*) and, as such, single gene knockout HAP1 pools were already available. Briefly, transfer vectors carrying sgRNAs of interest were packaged into lentiviruses as described above, scaling to a six-well plate format. Neat lentivirus-containing supernatant was used to transduce naïve HAP1 cells, which were selected for 72 hours in the presence of puromycin (2 μg/ml). Following a 7-day recovery and expansion phase, successful knockout was confirmed through targeted PCR amplification of extracted gDNA to isolate amplicons containing the intended CRISPR cut site, with PCR and sequencing primers designed through CRISPOR (*102*). Sanger sequencing of knockouts and NTCs were compared using Synthego ICE to determine knockout scores (*103*). sgRNA sequences and ICE results are presented in table S4. KO cell pools were used for venom challenge assays using resazurin reduction readouts as described above.

### Mice

All mice in this study were male C57BL/6 mice aged 8 to 15 weeks obtained from the Animal Resource Centre, WA, Australia. All experiments were approved by the Animal Ethics Committee at the University of Sydney under Approval 2094. Mice were housed in a specific pathogen–free facility on a 12-hour light-dark cycle, and standard chow, water, and enrichment were provided ad libitum. All in vivo behavioral assays were performed blind to treatment by a single female investigator. Assignment to treatment groups and injection was performed randomly by a separate investigator.

### Injections

Fifty micrograms of SNV in 25 μl of Hepes buffer (20 mM, Gibco) alone or preincubated with 60 μg of heparin (Sigma-Aldrich, no. H3393) was injected under isoflurane anesthesia (2 to 3% mixed with 1 liter of O_2_, IsoFlo Zoetis) into the mouse footpad. Mice were monitored until recovery from anesthesia.

### Behavioral testing

#### Spontaneous pain behavior

Following injection and recovery, mice were immediately placed into clear acrylic boxes on a raised acrylic table. Mouse behavior was recorded using a video camera (Panasonic) for 10 min. This was manually scored by a blinded female investigator for paw licking, which was the dominant immediate pain behavior.

#### Hargreaves thermal hyperalgesia assay

The Hargreaves test machine (Harvard Apparatus, no. 72-6692 (*104*)) was turned on 1 to 2 hours before recordings to ensure reliable infrared (IR) emissions. Mice were habituated in plexiglass boxes on a raised plexiglass table for 1 hour. The IR emitter/detector was placed directly underneath the center of each hind paw and initiated, recording the time from the start of IR emission until a paw withdrawal response. At minimum, three trials were performed per hind paw. Baseline withdrawal latency was established 1 week before injections and mice were tested at intervals between 1 and 48 hours after vehicle and SNV ± drug injections.

#### Von Frey mechanical allodynia assay

Mice were habituated in plexiglass boxes atop a wire mesh floor for 1 hour. The up-down Dixon method was used using a set of seven calibrated von Frey filaments (Semmes-Weinstein Filaments, North Coast Medical 0.02 to 1.0 g) on each hind paw in the sural territory (*105*). Filaments were applied to the hind paw until the fibers bowed and held for 3 s. Withdrawal of the hind paw within the 3-s period was counted as a positive response to the filament. Three trials were performed per hind paw. Baseline thresholds were established 1 week before injections and mice were again tested 7 hours after vehicle and venom ± drug injections.

### Statistics

Statistical analyses were performed using GraphPad Prism 9.2.0, GraphPad Software (San Diego, CA, USA; www.graphpad.com). A *P* value of 0.05 was considered statistically significant: ns, nonsignificant; *P < 0.05; **P < 0.01; ***P < 0.001; ****P < 0.0001.
