# Supplementary material for: Sea nettle jellyfish venom targets proteoglycans to cause cell death and pain
Source: Sci Adv. 2026 Sep 2;12(36):eaec7585. doi: 10.1126/sciadv.aec7585 (PMC13537241; doi:10.1126/sciadv.aec7585)
Supplement: Supplementary file 1 [file sciadv.aec7585_sm.pdf]

Science Advances
AAAS

Supplementary Materials for

Sea nettle jellyfish venom targets proteoglycans to cause cell death and pain

Christopher E. Denes *et al.*

Corresponding author: G. Gregory Neely, greg.neely@sydney.edu.au

Sci. Adv. **12**, eaec7585 (2026)
DOI: 10.1126/sciadv.aec7585

The PDF file includes:

Fig. S1
Legends for tables S1 to S5

Other Supplementary Material for this manuscript includes the following:

Tables S1 to S5

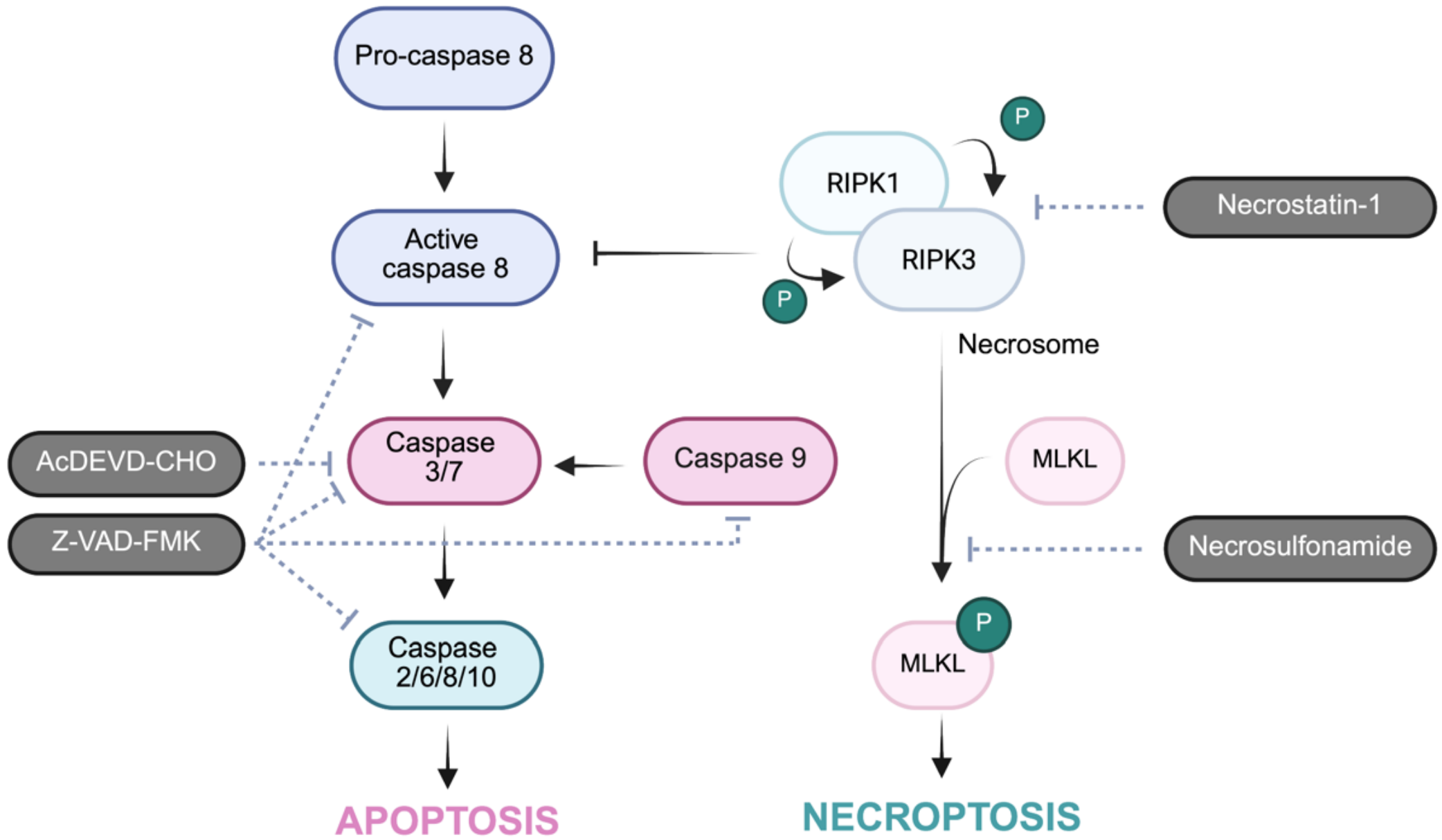

Fig. S1. Schematic representation of programmed cell death drug inhibitor targets. AcDEVD-CHO is a caspase 3- and caspase 7-targeting inhibitor of apoptosis. Z-VAD-FMK is a pan-caspase inhibitor. Necrostatin-1 and necrosulfonamide target separate elements of the necroptosis pathway. Created in BioRender. Neely, G. (2026) https://BioRender.com/qlhz9h6.

Other Supplementary Materials for this manuscript include the following:

Table S1. MAGeCKFlute output for 3-day SNV challenge screen.

Table S2. MAGeCKFlute output for 6-day SNV challenge screen.

Table S3. MAGeCKFlute output for 9-day SNV challenge screen.

Table S4. Single sgRNA HAP1 knockout cell pool knockout scores.

Table S5. Source data for non-volcano plots.
